# Supplementary material for: Exosomes Derived from Adipose Tissue-Derived Mesenchymal Stromal Cells Prevent Medication-Related Osteonecrosis of the Jaw through IL-1RA
Source: Int J Mol Sci. 2023 May 12;24(10):8694. doi: 10.3390/ijms24108694 (PMC10218172; doi:10.3390/ijms24108694)
Supplement: Supplementary file 1 [file ijms-24-08694-s001.zip › ijms-2243793-supplementary.pdf]

Article

Exosomes Derived from Adipose Tissue-Derived Mesenchymal Stromal Cells Prevent Medication-Related Osteonecrosis of the Jaw through IL-1RA

Yi Zheng [1,†], Xian Dong [1,2,†], Xinyu Wang [1], Jie Wang [1], Shuo Chen [1], Yang He [1], Jingang An [1], Linhai He [1,2,*] and Yi Zhang [1,*]

[1] Department of Oral and Maxillofacial Surgery, Peking University School and Hospital of Stomatology, Beijing 100081, China
[2] First Clinical Division, Peking University School and Hospital of Stomatology, Beijing 100081, China
* Correspondence: helinhai07@bjmu.edu.cn (L.H.); zhangyi2000@263.net (Y.Z.)
† These authors contributed equally to this work.

Table S1. The double-stranded sequence of siRNA from RiboBio company.

Product number	Sequence
stB0006799A	GACCAAATGTCAATTTAGA

Table S2. Primers of targeted gene.

mRNA	primer pairs (5′-3′)
Actin (h)	forward CATGTACGTTGCTATCCAGGC
	reverse CTCCTTAATGTCACGCACGAT
IL-1RA (h)	forward CATTGAGCCTCATGCTCTGTT
	reverse CGCTGTCTGAGCGGATGAA

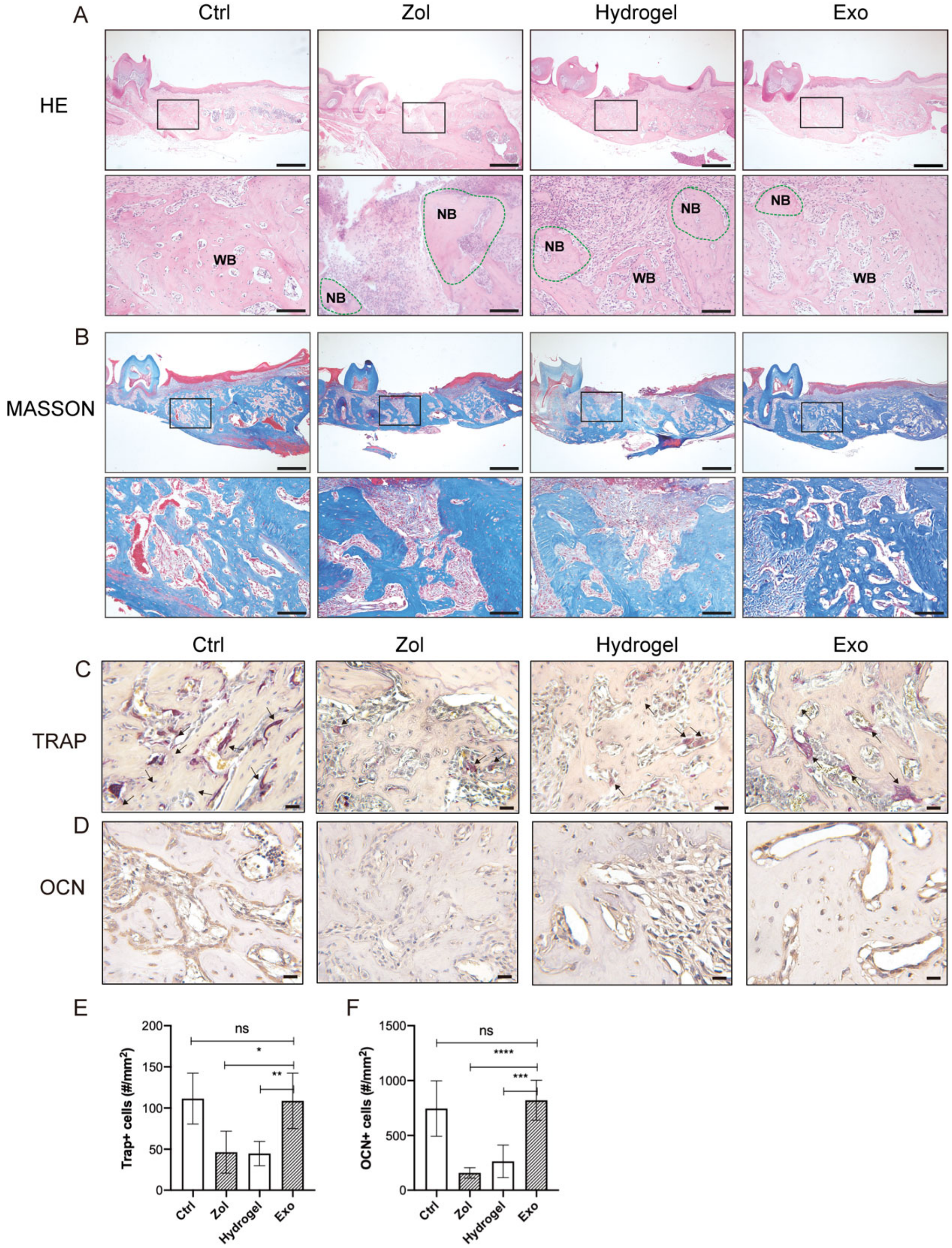

Figure S1. Exosomes might promote bone regeneration of tooth extraction. (A) Evaluation of 2 weeks after tooth extraction by H&E staining. Ctrl (non-drug treatment) group, Zol (treated with Zol) group, Hydrogel (treated with Zol and hydrogel) group, and Exo (treated with Zol, and hydrogel loaded with exosomes) group. Black square: areas were magnified. NB, necrotic bone; WB, woven bone. Scale bar=500 μm (upper), scale bar=100 μm (lower). **(B)** Evaluation of 2 weeks after tooth extraction by Masson's trichrome stained images. Black square: areas were magnified. Scale bar=500 μm (upper), scale bar=100 μm (lower). **(C)** Representative TRAP-stained images of tooth extraction sockets in each group. Black arrowhead: TRAP-positive cells. Scale bar=20 μm. **(D)** Images of OCN IHC staining of tooth extraction sockets in each group. Scale bar=20 μm. **(E, F)** Quantifying the number of TRAP-positive cells in the bone marrow and the expression of OCN in each group. (*p<0.05, **p<0.01, ***p<0.001, ****p<0.0001, ns: not significant).

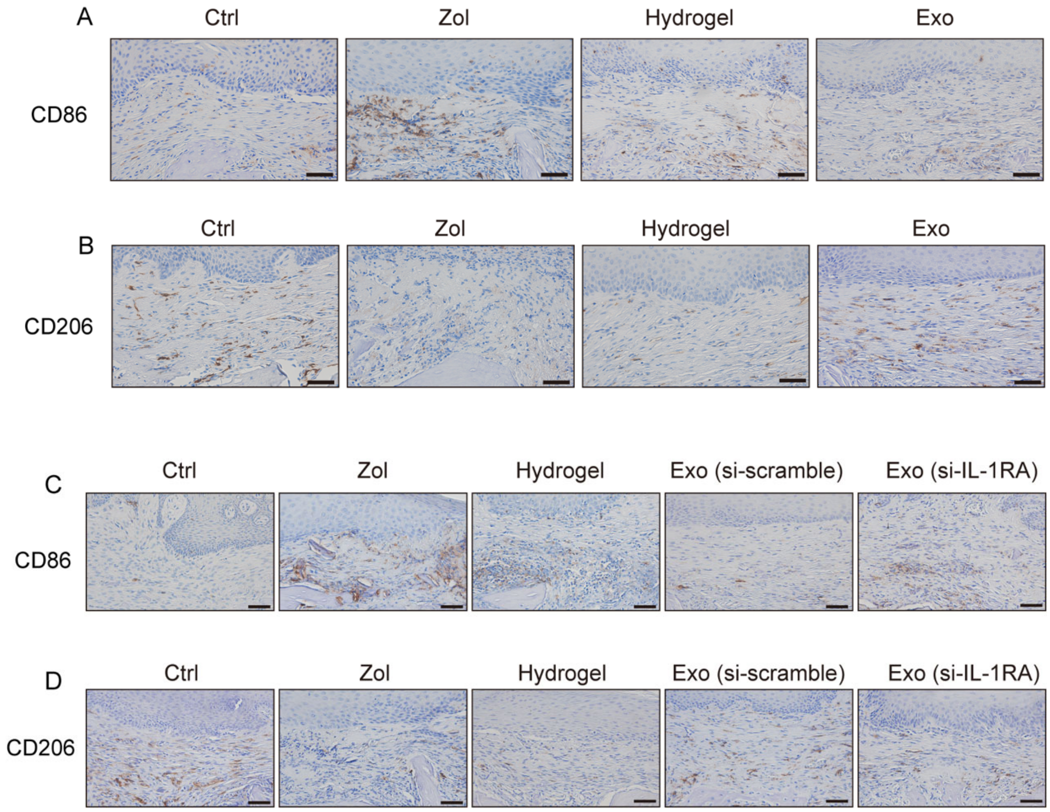

Figure S2. Exosomes decrease the numbers of CD86⁺ M1 macrophages and increase the numbers of CD206⁺ M2 macrophages, while knockdown of IL-1RA in MSC(AT)s-Exo attenuates the ability to promote gingival wound healing. (A) Images of CD86 IHC staining of gingiva tissue in each group [(Ctrl (non-drug treatment) group, Zol (treated with Zol) group, Hydrogel (treated with Zol and hydrogel) group, and Exo (treated with Zol, and hydrogel loaded with exosomes group)]. Scale bar=50 μm. (B) Images of CD206 IHC staining of gingiva tissue in each group. Scale bar=50 μm. (C) Images of CD86 IHC staining of gingiva tissue in each group [(Ctrl (non-drug treatment) group, Zol (treated with Zol) group, Hydrogel (treated with Zol and hydrogel) group, Exo (si-scramble) (treated with Zol, and hydrogel loaded with exosomes (si-scramble) group), and Exo (si-IL-1RA) group (treated with Zol and hydrogel loaded with exosomes (si-IL-1RA) group)]. Scale bar=50 μm. (D) Images of CD206 IHC staining of gingiva tissue in each group. Scale bar=50 μm.
